# Novel anthropometric indicators of visceral obesity predict the severity of hyperlipidemic acute pancreatitis

**DOI:** 10.1186/s12944-024-02112-1

**Published:** 2024-04-23

**Authors:** Yi Zhu, Yingbao Huang, Houzhang Sun, Lifang Chen, Huajun Yu, Liuzhi Shi, Weizhi Xia, Xuecheng Sun, Yunjun Yang, Hang Huang

**Affiliations:** 1 Department of Radiology, The First Affiliated Hospital of Wenzhou Medical University, Wenzhou, 325006 China; 2 Department of Hepatobiliary Surgery, The First Affiliated Hospital of Wenzhou Medical University, Wenzhou, 325006 China; 3 Department of Clinical Laboratory, The First Affiliated Hospital of Wenzhou Medical University, Wenzhou, 325006 China; 4 Department of Radiology, The Second Affiliated Hospital of Wenzhou Medical University, Wenzhou, 325006 China; 5 Department of Gastroenterology, The First Affiliated Hospital of Wenzhou Medical University, Wenzhou, 325006 China; 6 Department of Nuclear Medicine, The First Affiliated Hospital of Wenzhou Medical University, Wenzhou, 325006 China

## Abstract

**Background:**

Obesity substantially contributes to the onset of acute pancreatitis (AP) and influences its progression to severe AP. Although body mass index (BMI) is a widely used anthropometric parameter, it fails to delineate the distribution pattern of adipose tissue. To circumvent this shortcoming, the predictive efficacies of novel anthropometric indicators of visceral obesity, such as lipid accumulation products (LAP), cardiometabolic index (CMI), body roundness index (BRI), visceral adiposity index (VAI), A Body Shape Index (ABSI), and Chinese visceral adiposity index (CVAI) were examined to assess the severity of AP.

**Method:**

The body parameters and laboratory indices of 283 patients with hyperlipidemic acute pancreatitis (HLAP) were retrospectively analysed, and the six novel anthropometric indicators of visceral obesity were calculated. The severity of HLAP was determined using the revised Atlanta classification. The correlation between the six indicators and HLAP severity was evaluated, and the predictive efficacy of the indicators was assessed using area under the curve (AUC). The differences in diagnostic values of the six indicators were also compared using the DeLong test.

**Results:**

Patients with moderate to severe AP had higher VAI, CMI, and LAP than patients with mild AP (all *P* < 0.001). The highest AUC in predicting HLAP severity was observed for VAI, with a value of 0.733 and 95% confidence interval of 0.678–0.784.

**Conclusions:**

This study demonstrated significant correlations between HLAP severity and VAI, CMI, and LAP indicators. These indicators, particularly VAI, which displayed the highest predictive power, were instrumental in forecasting and evaluating the severity of HLAP.

## Introduction

Acute pancreatitis (AP) is a serious inflammatory condition with potentially fatal outcomes [1]. This condition often involves the inflammation of adjacent tissues and distant organ systems to varying extents [2]. Most AP cases in adults are attributed to gallstones or heavy alcohol use [3]. Approximately 20% of these cases may progress to severe acute pancreatitis (SAP), and approximately 20% of patients with SAP face life-threatening complications [4, 5]. Although critical care treatment has advanced in recent years, the mortality rate remains virtually unchanged [6].

The severity and prognosis of AP appear to vary by aetiology; however, most studies did not categorise aetiological patterns. Gallstone pancreatitis is caused by obstruction of the pancreatic duct by gallstones, and the resulting ductal hypertension and release of autonomously activated digestive enzymes lead to pancreatitis [7]. The underlying pathogenesis of hyperlipidemic acute pancreatitis (HLAP) is not fully understood and is not due to a single mechanism. Obstruction of pancreatic capillaries by large celiac particles is generally recognised to lead to hydrolysis of lipoproteins and release of free fatty acids (FFA), which are bound to serum albumin. The two polymers have detergent properties that increase intra-pancreatic blood viscosity and trigger an imbalance of intra-pancreatic acids and bases, leading to acidosis and subsequent transformation of inactive intra-alveolar trypsinogen into active trypsin, which results in pancreatic auto-digestion [8, 9]. The release of inflammatory mediators, such as IL-10, is increased in the pancreatic inflammatory response generated by FFA. These cytokines are critical in the development of HLAP. Although triglycerides themselves do not cause pancreatitis, pancreatic lipase has the ability to convert triglycerides into FFA, which can cause lipotoxicity [10, 11]. With improved living conditions and people placing greater importance on healthier lifestyles and better nutrition, HLAP now accounts for 10% of pancreatitis episodes [8] and is currently the third most common cause of AP worldwide [12]. Compared with AP caused by other aetiologies, HLAP exhibits higher severity, complication rates, and recurrence rates [13, 14]. Therefore, identifying the aetiology and assessing the severity of HLAP is important. Early prevention of SAP and related disorders enables patients to undergo more aggressive tests and treatments, including early fluid resuscitation and nutritional support, which are crucial for reducing the burden of care.

Abdominal obesity plays a critical role in the progression from AP to SAP [15]. According to a population-based study of more than 68,000 adults, abdominal obesity increased the risk of AP [16]. Therefore, it is worthwhile to study the use of common anthropometric indices, such as body mass index (BMI), to predict AP severity [17]. One drawback of this method is that it does not differentiate between muscle and fat accumulation [18–20]. The gold-standard imaging assessments of visceral adipose tissue (VAT), such as CT and MRI, have drawbacks including the high costs, radiation exposure, and time required. There is a growing trend towards the use of simple methods to assess visceral fat. Lipid accumulation products (LAP) [21], cardiometabolic index (CMI) [22], body roundness index (BRI) [23], visceral adiposity index (VAI) [12], A Body Shape Index (ABSI) [24], and Chinese visceral adiposity index (CVAI) [25] are relatively new and effective anthropometric indicators that have been studied to assess visceral obesity and compensate for the limitations of other methods. Most of these indicators have been primarily utilised and examined within the framework of metabolic syndrome (MetS), and their efficacy in predicting the severity of HLAP remains unexplored. This research aimed to explore the association between novel anthropometric indicators and HLAP severity and the prognostic role of these indicators in HLAP.

## Materials and methods

### Study population

Patients diagnosed with AP who were admitted to our hospital between July 2017 and April 2021 were included. Of the reviewed patients, 283 met the HLAP criteria below.

### Diagnostic criteria for HLAP

The diagnosis of AP adhered to the criteria established by the Atlanta International Symposium and revised Atlanta classification. These criteria include a) abdominal pain that was consistent with AP, characterised by a sudden onset of severe and persistent epigastric pain that may radiate to the back; b) serum lipase or amylase greater than three times the upper bound of the standard range; and c) presence of radiological findings that did not indicate cholelithiasis. The diagnosis of HLAP was confirmed when patients had a serum triglyceride (TG) level of 11.3 mmol/L or higher, or 5.65–11.3 mmol/L in the presence of celiac disease. The exclusion criteria for this study were as follows: a) individuals who consumed excessive alcohol prior to the onset of the condition or who had daily alcohol consumption exceeding 50 g for at least 5 years [26]; b) evidence of cholelithiasis detected by CT, MRI, or abdominal ultrasound; and c) patients aged < 18 years.

## Data collection

Sociodemographic characteristics, medical histories, and laboratory test results were collected from medical records. Professional researchers adhered to a standard protocol to measure the participants’ body height, waist circumference (WC), blood pressure, and weight. Baseline assessments involved collecting venous blood samples from all participants after a ≥ 8-hour all-night fast (no food or alcohol consumption, but water intake was allowed). Measurements included fasting blood glucose (FBG), creatinine (Cr), lactate dehydrogenase (LDH), total cholesterol (TC), total bilirubin (TBIL), high-density lipoprotein cholesterol (HDL-C), TG, low-density lipoprotein cholesterol (LDL-C), amylase, blood urea nitrogen (BUN), lipase, calcium (Ca), phosphorus (P), sodium (Na), potassium (K), and other biochemical parameters. A hepatobiliary and pancreatic surgeon with over 10 years of experience collected international rating scores such as Ranson, APACHE II, Marshall, and BISAP.

The formulae that were used are as follows:

$$\textrm{VAI}\left(\textrm{males}\right)=\left\{\textrm{WC}\left(\textrm{cm}\right)/\left(39.68+\left[1.88\times \textrm{BMI}\right]\right)\right\}\times \left(\textrm{TG}/1.03\right)\times \left(1.31/\textrm{HDL}\right)$$

$$\textrm{VAI}\ \left(\textrm{females}\right)=\left\{\textrm{WC}\left(\textrm{CM}\right)/\left(36.58+\left[1.89\times \textrm{BMI}\right]\right)\right\}\times \left(\textrm{TG}/0.81\right)\times \left(1.52/\textrm{HDL}\right)$$

$$\textrm{LAP}\ \left(\textrm{males}\right)=\left(\textrm{WC}\ \left(\textrm{cm}\right)-65\right)\times \textrm{TG}$$

$$\textrm{LAP}\ \left(\textrm{females}\right)=\left(\textrm{WC}\ \left(\textrm{cm}\right)-58\right)\times \textrm{TG}$$

$$\textrm{CVAI}\left(\textrm{males}\right)=-267.93+0.68\times \textrm{year}+0.03\times \textrm{BMI}+4.00\times \textrm{WC}\left(\textrm{cm}\right)+22.0\times \textrm{logTG}-16.32\times \textrm{HDL}-\textrm{C}$$

$$\textrm{CVAI}\ \left(\textrm{females}\right)=-187.32+1.71\times \textrm{year}+4.23\times \textrm{BMI}+1.12\times \textrm{WC}\left(\textrm{cm}\right)+39.76\times \textrm{logTG}-11.66\times \textrm{HDL}-\textrm{C}$$

$$\textrm{CMI}=\textrm{TG}/\textrm{HDL}-\textrm{C}\times \textrm{Waist}-\textrm{to}-\textrm{Height}\ \textrm{Ratio}\ \left(\textrm{WHtR}\right)$$

$$\textrm{ABSI}=\textrm{WC}\ \left(\textrm{m}\right)/\left(\textrm{BMI}\hat{\phantom{0}} \left(2/3\right)\times \textrm{height}\ \left(\textrm{m}\right)\hat{\phantom{0}} \left(1/2\right)\right)$$

$$\textrm{BRI}=364.2-365.5\times \textrm{sqrt}\ \left(1-\left(\textrm{WC}\left(\textrm{cm}\right)/\left(2\uppi \right)\right)\hat{\phantom{0}} 2/\left(0.5\times \textrm{height}\right)\hat{\phantom{0}} 2\right)$$

### Statistical analysis

IBM SPSS Statistics 18.0 was used for statistical analysis. The distribution of the data was checked for normalcy using the Kolmogorov–Smirnov test, and one-way ANOVA or Levene’s test was used to assess homogeneity of variance. For normally distributed continuous data, mean and standard deviation were measured; for skewed data, median and interquartile range were used. Categorical variables are expressed as percentages. Student’s t-test and non-parametric Mann–Whitney U-test were used to compare continuous data. Spearman’s rank correlation coefficients were used to examine the relationship between two continuous variables that were not normally distributed. Area under the curve (AUC) analysis was conducted to evaluate the diagnostic performance of novel anthropometric indicators in assessing the severity of AP. *P* < 0.05 was considered significant.

## Results

### Characteristics of participants

**Fig. 1:**
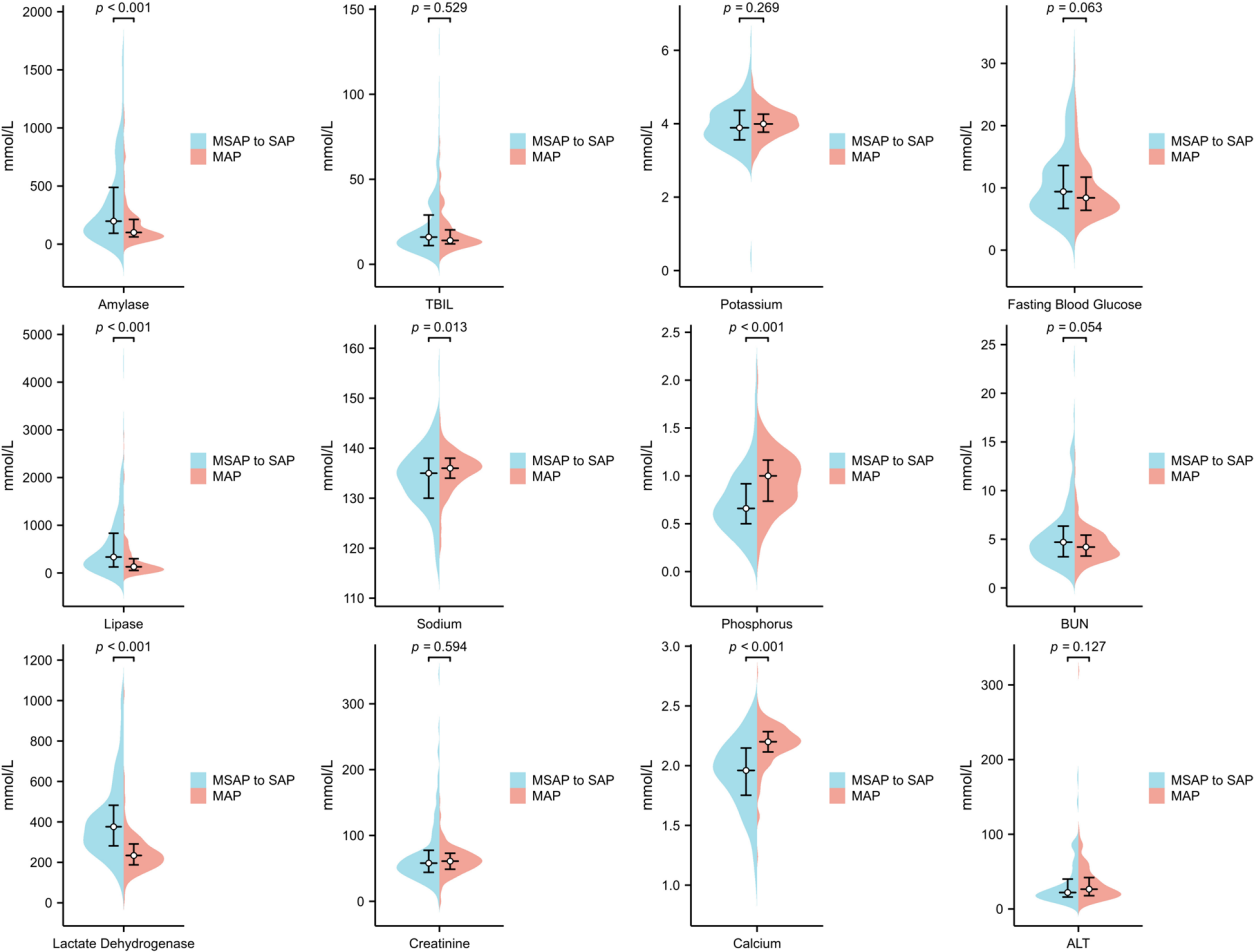
Correlation of laboratory tests with severity of HLAP. Abbreviations: ALT, alanine aminotransferase; BUN, blood urea nitrogen; MAP, mild acute pancreatitis, MSAP, moderately severe acute pancreatitis; SAP, severe acute pancreatitis; TBIL, total bilirubin

The data of 283 patients were retrospectively reviewed in this study. Patients diagnosed with moderately severe acute pancreatitis (MSAP) and SAP were grouped together. Participants were categorised based on AP severity as follows: 152 patients exhibited mild acute pancreatitis (MAP), 102 had MSAP, and 29 developed SAP. As shown in Table 1, there were 62 women (21.9%) and 221 men (78.1%). The average BMI was 25.71 kg/m^2^, the average WC was 88.73 cm, and the mean lipid level was 14.21. Participants were, on average, 40.7 ± 9.3 years old. The “MSAP to SAP” group had higher levels of LDH (373 vs. 234, *P* < 0.001), amylase (198 vs. 90, *P* < 0.001), and lipase (334 vs 130, *P* < 0.001) than the MAP group. Statistical analyses demonstrated that the severity of HLAP was associated with Ca, P, Na, lipase, amylase and LDH levels (Fig. 1).

**Table 1:** Basic characteristics of the participants

Variable	Total	MAP	MSAP to SAP	*P* value
Age (years)	40.73 ± 9.33	41.31 ± 8.51	40.05 ± 10.19	0.651
Gender (male)	221 (78.1%)	96 (43.4%)	125 (46.6%)	0.069
Hypertension	59 (20.8%)	31 (52.5%)	28 (47.5%)	0.840
Drinking	129 (45.6%)	67 (51.9%)	62 (48.1%)	0.584
WC (cm)	88.73 (83.27–96.06)	89.04 (83.86–95.17)	88.19 (82.18–97.16)	0.372
BMI (kg/m^2^)	25.71 (23.53–28.40)	25.90 (24.03–28.70)	25.53 (23.24–28.34)	0.204
TG (mmol/L)	14.21 (9.58–26.64)	12.26 (8.68–20.27)	18.96 (12.09–31.92)	<0.001
HDL-C (mmol/L)	0.61 (0.48–0.78)	0.68 (0.57–0.84)	0.52 (0.40–0.66)	<0.001
Diabetes	62 (39.5%)	29 (46.8%)	33 (53.2%)	0.402
LDH (mmol/L)	276 (209–388)	234.0 (186.0–282.0)	373.0 (278.0–489.0)	<0.001
TBIL (mmol/L)	15 (11–22)	14.0 (12.0–19.0)	16.0 (11.0–29.0)	0.528
ALT (mmol/L)	25 (17–44)	28.0 (18.0–46.0)	22.0 (16.0–44.0)	0.127
Amylase (mmol/L)	123 (70–295)	90 (57–201)	198 (92–500)	<0.001
Lipase (mmol/L)	203.5 (532.0–80.8)	130 (53–285)	334 (126–856)	<0.001
Creatinine (mmol/L)	61 (46–73)	62.0 (48.0–73.0)	58.0 (44.0–78.0)	0.594
BUN (mmol/L)	4.4 (3.2–5.8)	4.1 (3.2–5.3)	4.7 (3.2–6.4)	0.054
FBG (mmol/L)	8.6 (6.6–12.8)	8.5 (6.5–12.1)	9.0 (6.6–13.6)	0.090
K (mmol/L)	4.0 (3.6–4.3)	4.0 (3.7–4.2)	3.9 (3.5–4.4)	0.269
Na (mmol/L)	135 (132–138)	136.0 (134.0–138.0)	135.0 (131.0–138.0)	0.027
Ca (mmol/L)	2.1 (1.9–2.3)	2.2 (2.1–2.3)	2.0 (1.8–2.2)	<0.001
P (mmol/L)	0.8 ± 0.3	0.9 ± 0.3	0.7 ± 0.4	<0.001

*WC* waist circumference, *BMI* body mass index, *TG* triglyceride, *HDL-C* high-density lipoprotein cholesterol, *LDH* lactate dehydrogenase, *TBIL* total bilirubin, *ALT* alanine aminotransferase, *BUN* blood urea nitrogen, *FBG* fasting blood glucose

### Correlation between novel anthropometric indicators and laboratory parameters

**Fig. 2:**
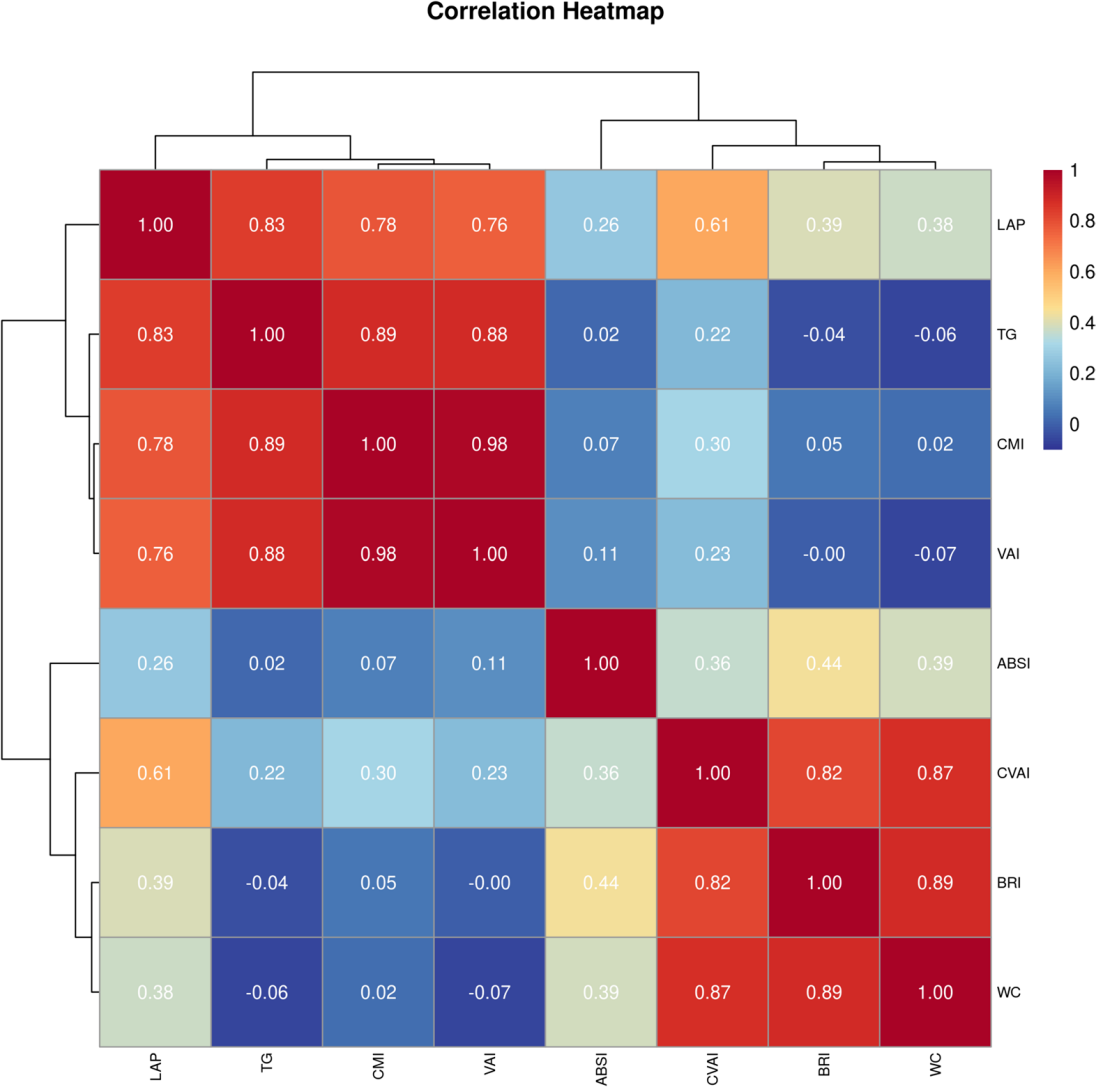
Correlation heatmap between anthropometric indicators of visceral obesity. Abbreviations: ABSI: a body shape index; BRI: body roundness index; CMI: cardiometabolic index; CVAI: Chinese visceral adiposity index; LAP: lipid accumulation products; TG: triglyceride; VAI: visceral adiposity index; WC: waist circumference

Figure 2 shows a significant positive correlation between TG and VAI, CMI, and LAP. Similarly, both BRI and CVAI showed a significant positive correlation with WC. A Statistically significant differences were observed between the increases in LAP and CMI (*r* = 0.783, *P* <

**Fig. 3:**
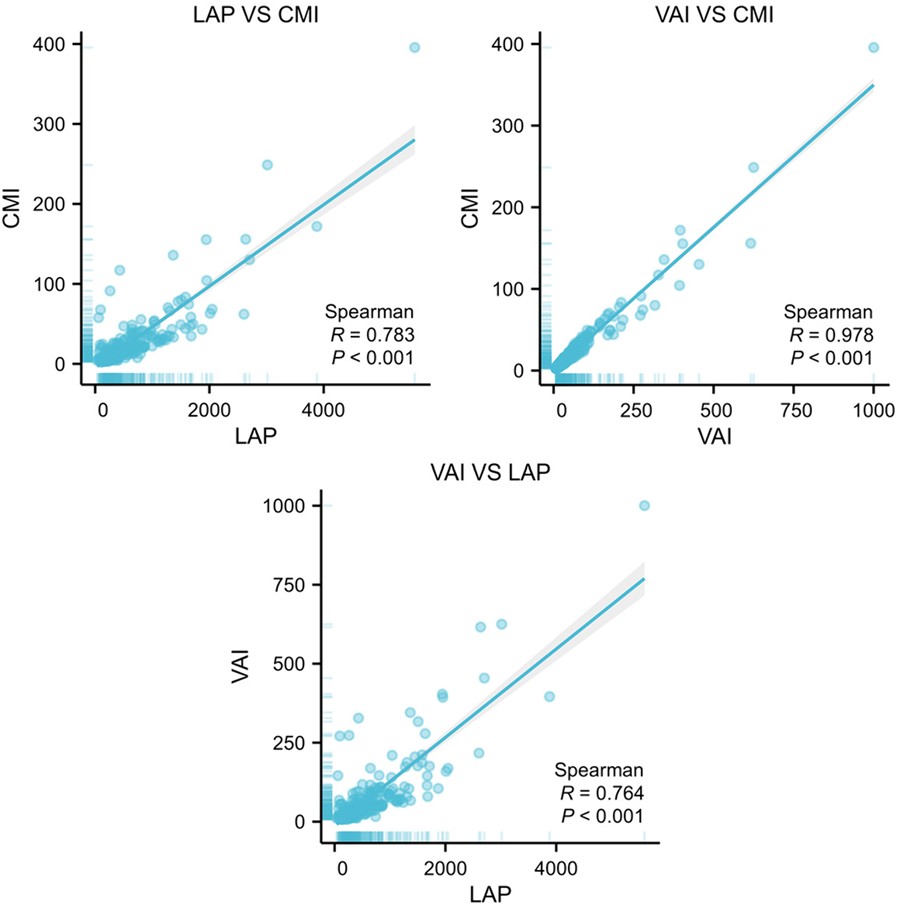
Correlation analysis between VAI, CMI, and CAP. Abbreviations: CMI: cardiometabolic index; LAP: lipid accumulation products; VAI: visceral adiposity index

0.001), VAI and CMI (*r* = 0.978, *P* < 0.001), and VAI and LAP (*r* = 0.764, *P* < 0.001) (Fig. 3).

### Comparison of novel anthropometric indicator characteristics between MAP group and “MSAP to SAP” group

As presented in Table 2, the “MSAP to SAP” group demonstrated a higher CMI (21.06 vs. 10.04, *P* = 0.002), VAI (52.46 vs. 26.50, *P* < 0.001), and LAP (444.41 vs. 318.33, *P* < 0.001) than the MAP group. No statistically significant difference was observed in other indicators (CVAI, BRI, and ABSI) between the two groups.

**Table 2:** Comparison of rating scales and body parameters for different severities of acute pancreatitis

Baseline variable	Total	MAP	MSAP to SAP	P value
SIRS	0.6 ± 0.5	0.82 ± 0.4	0.5 ± 0.5	<0.001
APACHE II	5.00 (3.00–8.00)	3.00 (1.00–5.00)	8.00 (6.00–10.00)	<0.001
BISAP	1.00 (0.00–2.00)	0.50 (0.00–1.00)	1.00 (1.00–2.00)	<0.001
Ranson	2.00 (1.00–3.00)	1.00 (1.00–2.00)	3.00 (2.00–4.00)	<0.001
Marshall	0.00 (0.00–1.00)	0.00 (0.00–0.00)	1.00 (0.00–2.00)	<0.001
LAP	366.82 (228.11–671.34)	318.33 (210.01–497.66)	441.41 (261.13–802.05)	0.002
CMI	14.25 (7.69–26.21)	10.04 (5.65–16.73)	21.06 (11.75–35.80)	<0.001
BRI	3.88 (3.34–4.78)	3.89 (3.40–4.77)	3.88 (3.25–4.78)	0.694
VAI	34.88 (19.67–68.75)	26.50 (14.75–44.73)	52.46 (32.19–95.23)	<0.001
ABSI	0.08 (0.08–0.08)	0.08 (0.08–0.08)	0.08 (0.08–0.08)	0.682
CVAI	134.14 (113.89–157.33)	132.87 (114.51–160.31)	136.15 (111.35–156.86)	0.801

*LAP* lipid accumulation products, *CMI* cardiometabolic index, *VAI* visceral adiposity index, *ABSI* a body shape index, *CVAI* Chinese visceral adiposity index, *BRI* body roundness index

### Ability of novel anthropometric indicators and international rating scale to predict HLAP severity

**Fig. 4:**
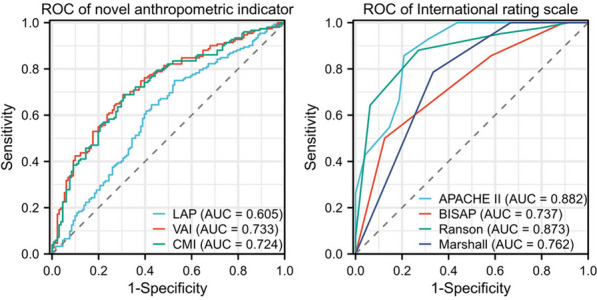
ROC curve of novel anthropometric indicators and international rating scales. Abbreviations: CMI: cardiometabolic index; LAP: lipid accumulation products; ROC: receiver operating characteristic; VAI: visceral adiposity index

The predictive performances of LAP, CMI, VAI, Marshall, APACHE II, Ranson, and BISAP scores for HLAP severity were compared using receiver operating characteristic (ROC) curves, as summarised in Table 3. The present study indicated that VAI achieved the best predictive performance with an AUC of 0.733 (95% confidence interval (CI): 0.678–0.784) among the three anthropometric indicators, closely followed by CMI at 0.724 (95% CI: 0.668–0.775) and LAP at 0.605 (95% CI: 0.548–0.665). Meanwhile, Ranson scores were associated with the intensity of HLAP (*P* < 0.001), and APACHE II demonstrated the highest AUC at 0.882 (95% CI: 0.797–0.941). Furthermore, the predictive probabilities of VAI (AUC = 0.733) and CMI (AUC = 0.724) closely parallelled the predictive power of BISAP (AUC = 0.737), attesting to the potential of novel anthropometric indicators in predicting the severity of HLAP, as shown in Fig. 4.

**Table 3:** Comparison of characteristics in predicting disease severity in HLAP

Variable	AUC	P value	95% CI
LAP	0.605	< 0.001	0.548 to 0.665
CMI	0.724	< 0.001	0.668 to 0.775
VAI	0.733	< 0.001	0.678 to 0.784
Marshall	0.762	< 0.001	0.661 to 0.845
APACHE_II	0.882	< 0.001	0.797 to 0.941
Ranson	0.873	< 0.001	0.786 to 0.934
BISAP	0.737	< 0.001	0.633 to 0.824

*LAP* lipid accumulation products, *CMI* cardiometabolic index, *VAI* visceral adiposity index

## Discussion

This study examined the correlation between anthropometric markers of visceral obesity and HLAP severity. We identified a significant association between these anthropometric indicators and the severity of AP, and demonstrated that VAI was the most accurate anthropometric predictor of HLAP. Notably, the number of patients with MSAP approached the number of patients with only MAP in this retrospective study. This may be because the study hospital is a large tertiary referral centre with a dedicated pancreatitis centre that accepts many patients with moderate to severe AP referred from outside institutions. Therefore, patients treated at the study hospital may present with higher disease severity than those in population-based studies. For the treatment of AP, the first principles of managing patients with mild AP include restriction of oral intake, enzyme inhibition, and intravenous rehydration with the use of lipid-modifying drugs such as fenofibrate capsules. In patients with MSAP and SAP, the primary goal of treatment is to preserve organ function by inhibiting pancreatic enzymes through the use of inhibitors to stabilise the internal environment, inhibit inflammatory damage, improve gastrointestinal motility, and reduce mortality by decreasing the incidence of early organ failure; in the later stages of disease, the main goals are to restore organ function and control infections and local complications [27]. Patients discharged from the hospital should receive long-term fibrate treatment to lower blood lipids and recommended lifestyle modifications to exercise more, control body weight, limit intake of fats and carbohydrates, and avoid alcohol consumption [28, 29].

AP has progressively become one of the most critical acute gastrointestinal diseases worldwide [30, 31]. Most AP cases are mild and do not require specific interventions. However, approximately 20% of patients are likely to develop SAP [32]. Current scoring systems, such as the Ranson score and APACHE II, seem to have realised their maximum potential in forecasting the severity of AP, but still exhibit low predictive accuracy [33]. No single scoring system has high accuracy in predicting different severity levels of pancreatitis or mortality in AP [34]. These scores often provide delayed predictive results (e.g. the Ranson score is standardised to 48 hours), are cumbersome to use (e.g. APACHE II includes more than 13 variables), or lack repeated validation (as is the case with most existing scores) [35]. Finally, none of the above scores consider obesity or visceral fat. However, underlying obesity is an important prognostic indicator [36]. As blood biochemistry tests and anthropometric measurements are routinely conducted in patients with AP, these parameters could be used to develop simple, dynamic, fast, and potentially predictive indicators.

HLAP is recognised to typically correlate with metabolic complications, such as obesity, which in turn increases the likelihood of elevated adipose tissue in patients with HLAP [37]. An abundance of clinical and experimental data indicates a robust association between obesity and AP severity [38, 39]. Current obesity-related markers aim to correlate body size with body fat and its distribution using numerical metrics, such as BMI. However, the discriminatory capability of BMI is suboptimal, as it fails to differentiate between adipose and fat-free tissues [18, 40]. Multiple studies have demonstrated that VAT outperforms BMI as the sole prognostic marker in patients with AP [15]. Given these observations, it is evident that a more comprehensive body index, that encompasses traditional anthropometric measurements, should consider body size. Several studies indicated that visceral obesity was linked to a poor prognosis of AP [41, 42]. Progress in anthropometrics has led to the development of new visceral obesity indices, such as LAP, VAI, CMI, CVAI, ABSI, and BRI. These were shown to represent the human body’s visceral fat content with relative accuracy, and each possesses unique properties for forecasting the risk of obesity-related diseases (such as MetS) [43–45]. A cross-sectional study of participants in the United States between 2017 and 2020 showed that an increased risk of developing osteoarthritis was associated with a higher LAP, showing a threshold effect. LAP that was less than 120.00 cm·mmol/L displayed an independent association with osteoarthritis [46]. Another retrospective study of 1498 patients found that increased VAI, a key indicator of insulin sensitivity and VAT function, was independently associated with cardiometabolic disease [47]. Wei et al. used the data of 5838 adults from the Chinese population and explored the predictive efficiency of CVAI for diabetes. CVAI showed a positive correlation with incidence of diabetes, and was found to be more effective when forecasting diabetes for Chinese individuals [48]. Liu et al. collected the anthropometric and biochemical data of 47,683 Chinese adults of normal weight and calculated CMI indicators; CMI was found to show good efficacy in recognizing the metabolically obese normal weight (MONW) phenotype [49]. A population-based cohort study repeatedly measured BRI, calculated its dynamic trajectory, and used Cox proportional risk models to analyse the association between BRI trajectory and cardiovascular disease events. BRI trajectory was observed to significantly correlate with the risk of cardiovascular disease, and the association was more significant in young people [50]. A cross-sectional study of 5245 people with acentric cerebrovascular disease showed that ABSI was independently correlated with carotid plaques and was not affected by sex. As ABSI levels increased, the prevalence of atherosclerosis increased linearly [51].

In this study, the correlations among novel anthropometric indicators of visceral obesity, VAI, LAP, CMI, ABSI, CVAI, BRI, and HLAP severity, were examined. Consistent with previous studies, Ji et al. discovered that visceral adipose tissue serves as a beneficial indicator of HLAP severity and prognosis [41]. Similarly, Natu et al. concluded that amplification in the VAT area corresponds to an increase in the severity of AP, as measured by the total cross-sectional area of the VAT. These measurements were taken using CT at the lumbar L2-L3 intervertebral disc space in males and the lumbar L3-L4 intervertebral disc space in females [52]. However, a drawback of these studies is that the underlying region of the VAT could be masked by subcutaneous and intra-abdominal oedema, potentially resulting in underestimation of fat distribution measurements. Despite this, ABSI and BRI, although novel anthropometric indicators of visceral obesity, failed to exhibit a significant correlation with HLAP severity, potentially owing to race variations. These indices were initially developed in Western countries (specifically in the United States) [53]. As Asians typically have a considerably lower BMI than their Western counterparts (the difference is approximately 2–3 kg/m^2^ for age- and sex-matched individuals) [54], these indices should be adjusted according to findings from other studies [55, 56] to ensure suitability for application in different populations with distinct characteristics.

### Study strengths and limitations

In general, this retrospective study revealed that these novel anthropometric indicators of visceral obesity, which are fast, simple, and dynamically monitorable methods, could predict the severity of HLAP. In addition, this research was the first to compare anthropometric indicators of visceral obesity with other clinical scoring systems in AP and to show comparable predictive accuracy with traditional scoring systems. When set against the existing international rating scale, the predictive efficacies of VAI, LAP, and CMI do not match with those of APACHE II and Ranson, but are roughly equivalent to those of Marshall and BISAP. In addition, these novel indicators may serve as a low-cost surrogate method for early prediction of the severity in specific patients (e.g., those for whom clinical scores cannot be obtained due to physical factors), which can provide better care (e.g., early enteral nutrition and maintenance of organ function).

This retrospective study has several limitations. First, only a limited number of patients with HLAP were studied, which imposed a constraint on the sample size for analysis. Retrospective study samples from a single institution may lead to selection bias. Second, it is possible that some patients experienced persistently increasing TG levels due to underlying AP, which may further increase the heterogeneity of the patient population arising from the retrospective nature of this study. Third, in local clinical practice, there is an absence of genetic testing to exclude patients with hereditary pancreatitis and familial hypercholesterolaemia from the retrospective study, which may further affect the predictive power of visceral obesity on HLAP. Finally, as the results were drawn from a single institution, they should be interpreted with caution, and a more comprehensive, multi-centre study involving a larger participant pool should be conducted to examine the true predictive value of visceral obesity indicators.

## Conclusions

The present study indicates notable associations between HLAP severity and VAI, CMI, and LAP. Amongst these anthropometric indicators, VAI demonstrated the highest predictive efficacy for HLAP severity. The findings present a rapid, straightforward, cost-effective, and dynamically trackable approach for predicting HLAP severity.

## Abbreviations

HLAP: Hyperlipidemic acute pancreatitis
LAP: Lipid accumulation products
CMI: Cardiometabolic index
VAI: Visceral adiposity index
ABSI: A body shape index
CVAI: Chinese visceral adiposity index
BRI: body roundness index
